# Subclinical Hypothyroidism among Patients with COVID-19 Infection in a Tertiary Care Centre: A Descriptive Cross-sectional Study

**DOI:** 10.31729/jnma.8187

**Published:** 2023-06-30

**Authors:** Prabin Adhikari, Rasu Singh

**Affiliations:** 1 Department of Internal Medicine, Nepal Medical College and Teaching Hospital, Jorpati, Kathmandu, Nepal

**Keywords:** *COVID-19*, *hypothyroidism*, *thyroid gland*

## Abstract

**Introduction:**

Hypothyroidism occurs as a consequence of chronic autoimmune inflammation of the thyroid gland, which occurs due to the reduced function in the secretion of thyroid hormones. The coronavirus disease infection has shown many complications in all organic systems, during the acute phase of infection and in the post-COVID-19 period. SARS-CoV-2 may induce thyroid dysfunction that is usually reversible, including subclinical and atypical thyroiditis. The aim of this study was to find out the prevalence of subclinical hypothyroidism among patients with COVID-19 infection in a tertiary care centre.

**Methods:**

A descriptive cross-sectional study was conducted in the Department of Internal Medicine of a tertiary care centre from 1 September 2022 to 28 February 2023 after obtaining ethical approval from the Research and Institutional Review Committee (Reference number: 15-079/080). Convenience sampling method was used. Point estimate and 95% Confidence Interval were calculated.

**Results:**

Among 38 patients with COVID-19, subclinical hypothyroidism was seen among 23 (60.53%) (44.99-76.07, 95% Confidence Interval).

**Conclusions:**

The prevalence of subclinical hypothyroidism among COVID-19 patients was found to be similar to other studies done in similar settings.

## INTRODUCTION

Subclinical hypothyroidism (SCH), also called mild thyroid failure, is diagnosed when peripheral thyroid hormone levels are within normal reference laboratory range but serum thyroid-stimulating hormone (TSH) levels are mildly elevated. This condition occurs in 3% to 8% of the general population. It is more common in women than men, and its prevalence increases with age. The most common cause of hypothyroidism including subclinical hypothyroidism is autoimmune in nature.^1^

Coronavirus disease 2019 (COVID-19) affects the respiratory system mainly.^2^ There have been several studies that COVID-19 infection results in endocrinopathies.^3-6^ The thyroid gland tissue with high angiotensin-converting enzyme 2 expression, has been associated with coronavirus infection.^6^ There is currently inadequate data regarding COVID-19's impact on the thyroid. Available studies were done during the active phase of COVID-19 or included patients admitted to intensive care units.

The aim of this study was to find out the prevalence of subclinical hypothyroidism among patients with COVID-19 infection in a tertiary care centre.

## METHODS

This descriptive cross-sectional study was done in the Department of Internal medicine at Nepal Medical College Teaching Hospital (NMCTH) from 1 September 2022 to 28 February 2023 after obtaining ethical approval from the Research and Institutional Review Committee of the NMCTH (Reference number: 15-079/080). Patients who gave written consent were included in this study. Patients who were treated previously for thyroid dysfunction before COVID-19 were excluded from the study. Convenience sampling method was used. The sample size was calculated using the following formula:


$$\begin{array}{ll} \text{n} & ={\text{Z}}^{2}\times \frac{\text{p}\times \text{q}}{{\text{e}}^{\text{2}}}\\ & =1.96^{2}\times \frac{0.067\times 0.933}{0.10^{2}}\\ & =24 \end{array}$$

Where,

n = minimum required sample size
Z = 1.96 at 95% Confidence Interval (CI)
p = prevalence of thyroid dysfunction among COVID-19 taken from previous study, 6.7%^7^
q = 1-p
e = margin of error, 10%

The total size calculated was 24. However, the final sample size taken was 38.

Patients were included in this study, only after they gave written consent for history taking, physical examination, and necessary investigations. Basic data including age, gender, occupation, history of COVID-19 infection, and family history of thyroid dysfunction were taken from all the participants in the study. Body mass index (BMI) was measured from all participants. COVID-19 patients were categorized as per World Health Organisation (WHO) clinical COVID-19 disease severity classification.^1^ Subclinical hyperthyroidism is defined as normal serum-free thyroxine (T4) and triiodothyronine (T3) concentrations in the presence of a subnormal TSH (<0.5 mU/l). The venous blood sample was collected and sent for T3, T4 and TSH. For primary hypothyroidism, the T3 and T4 level lower than the normal range (3.32-7.62 pmol/l) & (8.8-23.6 pmol/l) respectively were taken and primary hyperthyroidism was characterized by the higher level of T3 (>7.62 pmol/l) and T4 (>23.6 pmol/l) and low level of thyroid stimulating hormones (<0.4 mIU/l). For subclinical hypothyroidism, T3 and T4 levels are in the normal ranges (3.32-7.62 pmol/l) & (8.8-23.6 pmol/l) respectively but the TSH levels exceed the normal range of 0.45-5.32 pmol/l.

Data were entered in an Excel sheet and analyzed using IBM Statistics SPSS 20. Point estimate and 95% CI were calculated.

## RESULTS

**Figure 1:**
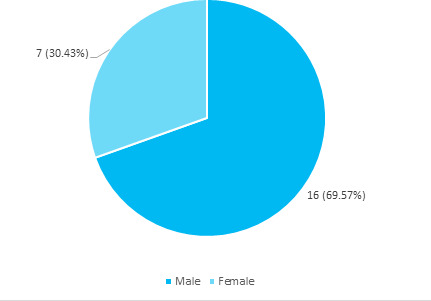
Gender distribution among patients with subclinical hypothyroidism (n= 23).

Among 38 patients with COVID-19, subclinical hypothyroidism was seen in 23 (60.53%) (44.9976.07, 95% CI). The median age of the population with subclinical hypothyroidism is 48 years. TSH of more than 10 was detected in 11 (47.83%) among patients with subclinical hypothyroidism. The majority of the patients with subclinical hypothyroidism 16 (69.57%) were males whereas 7 (30.43%) were female (Figure 1).

Among patients with subclinical hypothyroidism, COVID-19 severity was mild among 15 (65%), followed by 7 (30%) with moderate and 1 (5%) had severe disease (Table 1).

**Table 1:** Clinical COVID-19 severity grading with subclinical hypothyroidism (n = 23).

Clinical COVID-19 severity grading	n (%)
Mild	15 (65)
Moderate	7 (30)
Severe	1(5)

A total of 11 (48%) patientswith subclinicalhypothyroidism had BMI more than 25 (Table 2).

**Table 2:** BMI among patients with subclincal hypothyroidism (n= 23).

BMI	n (%)
<23	8 (35)
23-25	4 (17)
>25	11 (48)

## DISCUSSION

The prevalence of subclinical hypothyroidism in our study was 23 (60.53%). A hospital-based study reported a prevalence of 20.42%, which is more than double compared to other populations (1.4-7.8%).^8^ A recent study concluded that COVID-19 susceptibility and its severity might increase the risk of hypothyroidism including subclinical hypothyroidism.^9,10^ This is similar to another study conducted in Bosnia, where they found a significant difference in the number of patients with hypothyroidism and subclinical hypothyroidism in 2020 and 2021 in comparison to 2019.^11^ About 78.3% of patients had subclinical hypothyroidism 2 months post-COVID-19 infection in those with thyroid dysfunction which the persisted in 33.6% of the subjects of the patients after one month of follow up which was higher compared to our study.^12^ Several studies have identified cases of COVID-19-related primary hypothyroidism. However, they have cautioned the timing of thyroid function tests following COVID-19 and the use of glucocorticoid in the treatment could have an impact on the results including family history and the presence of autoantibodies.^3,4^ In contrast to this prospective study another prospective study did not find any alterations in thyroid hormone levels three months after SARS-CoV-2 infection in the cohort with subclinical hypothyroidism.^13^ In yet another study has demonstrated a significant prevalence of subclinical hypothyroidism in COVID-19 infected patients with a family history of thyroid disease, and/or a high level of thyroid peroxidase (TPO) antibodies.^14^

In contrary to our finding, several other studies have found hyperthyroidism in COVID-19 patients. A similar study found that the serum concentrations of TSH and total T3 were considerably lower in patients with COVID-19 than in a control group.^5^ Another study of hospitalized COVID-19 patients conducted in Hong Kong showed that the majority of those with abnormal thyroid function (13.1% of the group) had low TSH concentrations, but only one of 191 participants (0.5%) had a high TSH concentration and a high TPO antibody titre.^6^ Abnormal thyroid function was common in patients with COVID-19, particularly hyperthyroidism in another study.^7,15^

The patients with hypothyroidism and subclinical hypothyroidism were more likely to be female.^16^ The reasons for this gender disparity in the prevalence of thyroid disorders are not completely understood. In our study, however, subclinical hypothyroidism was more common among males with COVID-19. Similarly, more men than women had abnormal thyroid function tests (64% vs. 36%) in another study of COVID-19 patients.^17^

In our study, among patients with subclinical hypothyroidism 1 (5%) had severe COVID-19 infection and mild infection was seen in 15 (65%) of cases. Regardless of disease severity, recently a growing number of studies have been reported in the literature that patients may have other comorbidities weeks and months after the onset of COVID-19 including thyroid dysfunction.^18,19^

A BMI of more than 25 was seen in 11 (48%) subclinical hypothyroidism cases. Both thyroid dysfunction and higher BMI have been linked with longer disease morbidly in literature.^15,20,21^ Apart from high BMI no other risk factors were found that could be linked with subclinical hypothyroidism in our study. This too could also be a confounding factor as thyroid dysfunction particularly subclinical hypothyroidism has been reported to be associated with obesity.^22,23^

The limitation of our study is that the study was carried out among a small sample size and in a single centre, thus cannot be generalized to the general population. There were several confounding factors which could not be fully excluded from the descriptive study. Multicentre, longitudinal studies looking into this aspect are needed for further insights into thyroid dysfunction status.

## CONCLUSIONS

The prevalence of subclinical hypothyroidism among COVID-19 patients was found to be similar to other studies done in similar settings. Therefore, this indicates that these patients need to be evaluated thoroughly for thyroid dysfunction and this information should be kept in mind while evaluating these patients
